# Hypotonic Swelling Method for the Isolation of Pure Mitochondria From Primary Human Skeletal Myoblasts for Proteomic Studies

**DOI:** 10.1111/jcmm.70370

**Published:** 2025-01-20

**Authors:** Evrim Aksu‐Menges, Eray Taha Kumtepe, Gurler Akpinar, Burcu Balci‐Hayta

**Affiliations:** ^1^ Department of Medical Biology, Faculty of Medicine Hacettepe University Ankara Turkey; ^2^ Department of Medical Biology, Faculty of Medicine Kocaeli University Kocaeli Turkey

**Keywords:** differential centrifugation, hypotonic swelling method, mitochondria isolation, primary human skeletal myoblasts

## Abstract

Mitochondria play a fundamental role in energy metabolism, particularly in high‐energy‐demand tissues such as skeletal muscle. Understanding the proteomic composition of mitochondria in these cells is crucial for elucidating the mechanisms underlying muscle physiology and pathology. However, effective isolation of mitochondria from primary human skeletal muscle cells has been challenging due to the complex cellular architecture and the propensity for contamination with other organelles. Here, we compared four different methods to isolate mitochondria from primary human skeletal myoblasts regarding total protein yield, mitochondrial enrichment capacity and purity of the isolated fraction. We presented a modified method that combines differential centrifugation with a hypotonic swelling step and a subsequent purification process to minimise cellular contamination. We validated our method by demonstrating its ability to obtain highly pure mitochondrial fractions, as confirmed by Western Blot with mitochondrial, cytosolic and nuclear markers. We demonstrated that proteomic analysis can be performed with isolated mitochondria. Our approach provides a valuable tool for investigating mitochondrial dynamics, biogenesis and function in the context of skeletal muscle biology in health and disease. This methodological advancement opens new avenues for mitochondrial research and its implications in myopathies, sarcopenia, cachexia and metabolic disorders.

## 1 Introduction

In the context of skeletal muscle cells, mitochondria are crucial for cellular function, regeneration and adaptation to stress; this makes them a focal point of interest in understanding muscle physiology and pathology [1, 2, 3]. Mitochondrial pathology is generally studied in primary mitochondrial myopathies, such as Leigh syndrome, and mitochondrial encephalomyopathy, lactic acidosis and stroke‐like episodes (MELAS) which are caused by mutations in genes encoding OXPHOS machinery proteins. However, alterations in mitochondrial protein expression profile are also implicated in neuromuscular diseases, cancer, autism and systemic conditions. For instance, in obesity, mitochondria in skeletal muscle often exhibit reduced oxidative capacity and impaired dynamics, contributing to insulin resistance [4, 5]. Mitochondrial proteomics, a comprehensive study of proteins within mitochondria, provides valuable information about the molecular mechanisms underlying muscle health, disease and ageing [6, 7, 8]. However, the accuracy and depth of proteomic analysis depend on the purity of the isolated mitochondria. Thus, the choice of isolation method is a critical step that can profoundly influence the outcomes of proteomic studies [9].

To date, various methods have been developed to isolate mitochondria, each with its advantages and limitations. Besides the utilisation of commercially available isolation kits, techniques such as differential and density‐gradient centrifugation are frequently used to isolate mitochondria [10, 11, 12]. With such techniques, pure mitochondria isolation can be successfully performed in tissues and immortalised cell lines [13, 14, 15]. Since the choice of source material for mitochondrial isolation is related to the energy demands of the tissue, brain, liver and skeletal muscle are preferred for isolation due to their higher mitochondrial content [10, 12, 16]. Primary skeletal muscle cells are more relevant biological tools than genetically and phenotypically unstable immortalised cell lines. They offer a more accurate and adaptable platform for modelling chronic diseases, such as neuromuscular diseases, obesity and cachexia because they reflect the genetic background of the primary tissues from which they are derived and mimic the disease state very well in vitro [17, 18, 19]. However, the use of primary skeletal muscle cells for mitochondria isolation remains a rarity. This is largely due to the challenges associated with isolating pure mitochondria from these cells, such as the limited number of cells due to replicative senescence [20], which complicates the isolation process. There is only one known study that has attempted such isolation without subsequent verification of the purity of the mitochondrial fraction [21].

This paper presents a comparative analysis of four distinct methods for isolating mitochondria from primary human skeletal myoblasts. We hypothesized that different mitochondria isolation methods would have different efficiencies in obtaining pure mitochondria and aimed to identify the most suitable technique that allows the purest of mitochondrial fraction for reliable proteomic analyses. These include two commercially available isolation kit protocols and two manual techniques (differential centrifugation and hypotonic swelling). Our investigation not only focuses on the protein yield of the isolated mitochondria but also provides a purity check framework to validate the efficacy of each method. Establishing a robust protocol that facilitates using primary human skeletal myoblasts in mitochondrial research may improve our understanding of mitochondrial function and dysfunction in human health and disease.

## 2 Methods

### 2.1 Cell Culture

In this study, the experimental protocol was approved by the Hacettepe University Faculty of Medicine Ethical Review Board (2020/07‐12). Control primary human skeletal myoblasts that were isolated from the quadriceps muscle biopsy sample (Sample ID: 8063, male, age at biopsy: 2 and NH11‐1462A, female, age at biopsy: 4) with no diagnostic pathology were supplied by the MRC Centre for Neuromuscular Disease Biobank London (REC reference number 06/Q0406/33). Primary human skeletal myoblasts were proliferated in Skeletal Muscle Cell Growth Medium (PromoCell) supplemented with Supplement mix (PromoCell), and 2 mM L‐glutamine in an incubator at 37°C with 5% CO_2_. The cells were passaged upon reaching 70%–80% confluence. An equal number of cells at passage numbers 3–5 (3 T75 flasks, around 9 million cells) were used for each mitochondrial isolation method.

### 2.2 Kit‐Based Mitochondrial Isolation Methods

Mitochondrial isolation using two different commercially available kits (Qproteome Mitochondria Isolation Kit (Qiagen) and Mitochondria Isolation Kit for Cultured Cells (Thermo Scientific)) was applied according to the manufacturer's instructions. Both the long and short methods were applied as the Qproteome Mitochondria Isolation Kit (Qiagen) recommended. However, for the Mitochondria Isolation Kit for Cultured Cells (Thermo Scientific), mitochondrial isolation was performed with 70 strokes (up and down) of tight pestle in a hand‐held Dounce homogeniser. Buffer compositions of the kits are not provided by the manufacturers.

### 2.3 Isolation of Mitochondria by Differential Centrifugation

Cells grown in three T75 flasks were washed with 1× PBS and removed from the flasks using a cell scraper in 2 mL of ice‐cold PBS and transferred to 15 mL tubes. 3 mL of cold PBS was added to the flask to collect the remaining cells, which were then transferred to the same tube. The cells were centrifuged at 4°C at 600 × *g* for 5 min. After centrifugation, the supernatant was removed, and the cell pellet was resuspended in Isolation buffer‐1. 20 strokes (up and down) of tight pestle lysed the cells in an ice‐cold Dounce homogeniser. The cell suspension was incubated on an end‐over‐end shaker for 30 min at 4°C, followed by centrifugation at 800 × *g* for 15 min at 4°C to precipitate the cell debris, nuclei, and unbroken cells. After centrifugation, the supernatant was transferred to a new tube and centrifuged again at the same speed for 10 min at 4°C. The supernatant, thoroughly cleared of cell debris, was transferred to a separate tube and centrifuged at 18,000 × *g* for 10 min at 4°C to precipitate the mitochondrial fraction. After centrifugation, the supernatant was removed, and the pellet was resuspended in 100 μL of Isolation buffer‐1 and centrifuged at 18,000 × *g* for 10 min at 4°C. Following centrifugation, the supernatant was removed, and the pellet containing mitochondria was resuspended in SOL buffer. The buffers used in mitochondrial isolation are provided in Table 1. The mitochondrial fraction was sonicated 3 times with 10 s pulses and 30 s pauses at 20 kHz and 20% amplitude, and mitochondrial proteins were quickly frozen in liquid nitrogen and stored at −80°C.

**TABLE 1:** The buffers used in differential centrifugation and hypotonic swelling method.

	Isolation buffer‐1	Isolation buffer‐2	Hypotonic buffer	Hypertonic buffer
Isolation buffer	50 mM Tris–HCl pH 7.4 0.075 M sucrose 0.225 M mannitol 0.1 M KCl 0.2% Fatty acid‐free BSA Protease inhibitor cocktail Phosphatase inhibitor	10 mM MOPS, pH 7.2 0.225 M sucrose 0.075 M mannitol 1 mM EGTA Protease inhibitor cocktail Phosphatase inhibitor	10 mM MOPS pH 7.2 100 mM sucrose 1 mM EGTA Protease inhibitor cocktail Phosphatase inhibitor	10 mM MOPS pH 7.2 1.25 M sucrose
SOL buffer	50 mM Tris–HCl pH 6.8			
(Mitochondria resuspension buffer)	1 mM EDTA 0.5% Triton X‐100 Protease inhibitor cocktail Phosphatase inhibitor			

### 2.4 Isolation of Mitochondria by Hypotonic Swelling

**FIGURE 1:**
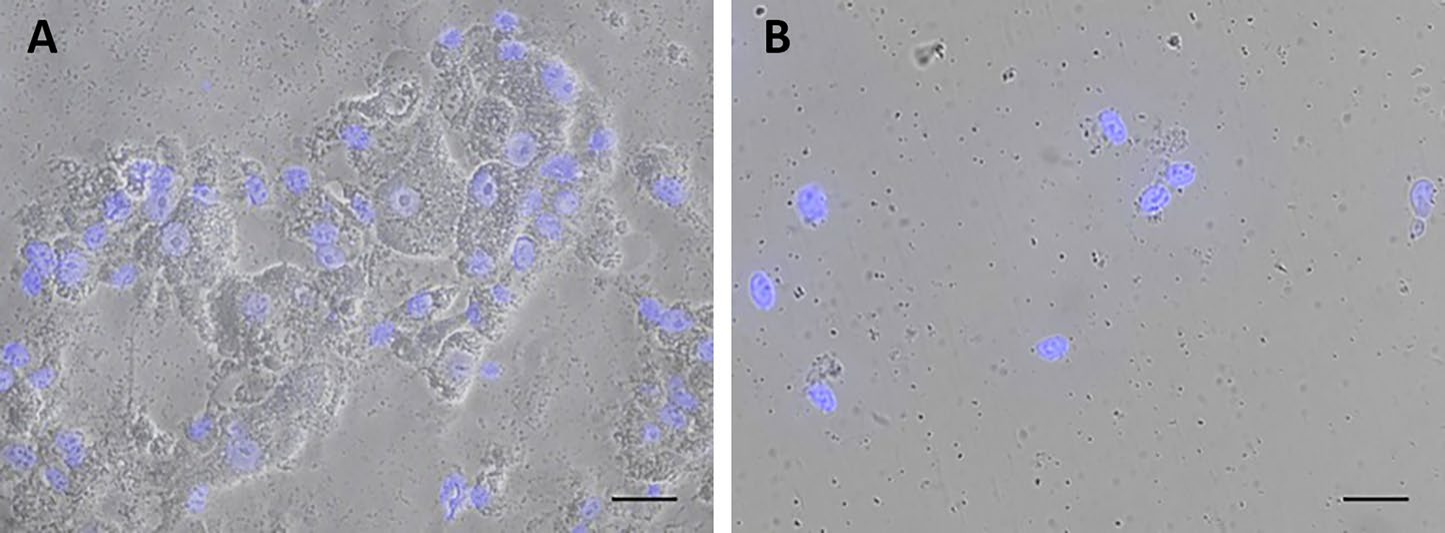
(A) Phase‐contrast images showing the morphology of the cells during the hypotonic swelling method. The cells were shown to be swollen after incubation in the hypotonic buffer. (B) After homogenization, the cell membrane was disrupted, but the nuclei remained intact. Nuclei were counterstained with DAPI (blue). Scale bars: 50 μm.

**FIGURE 2:**
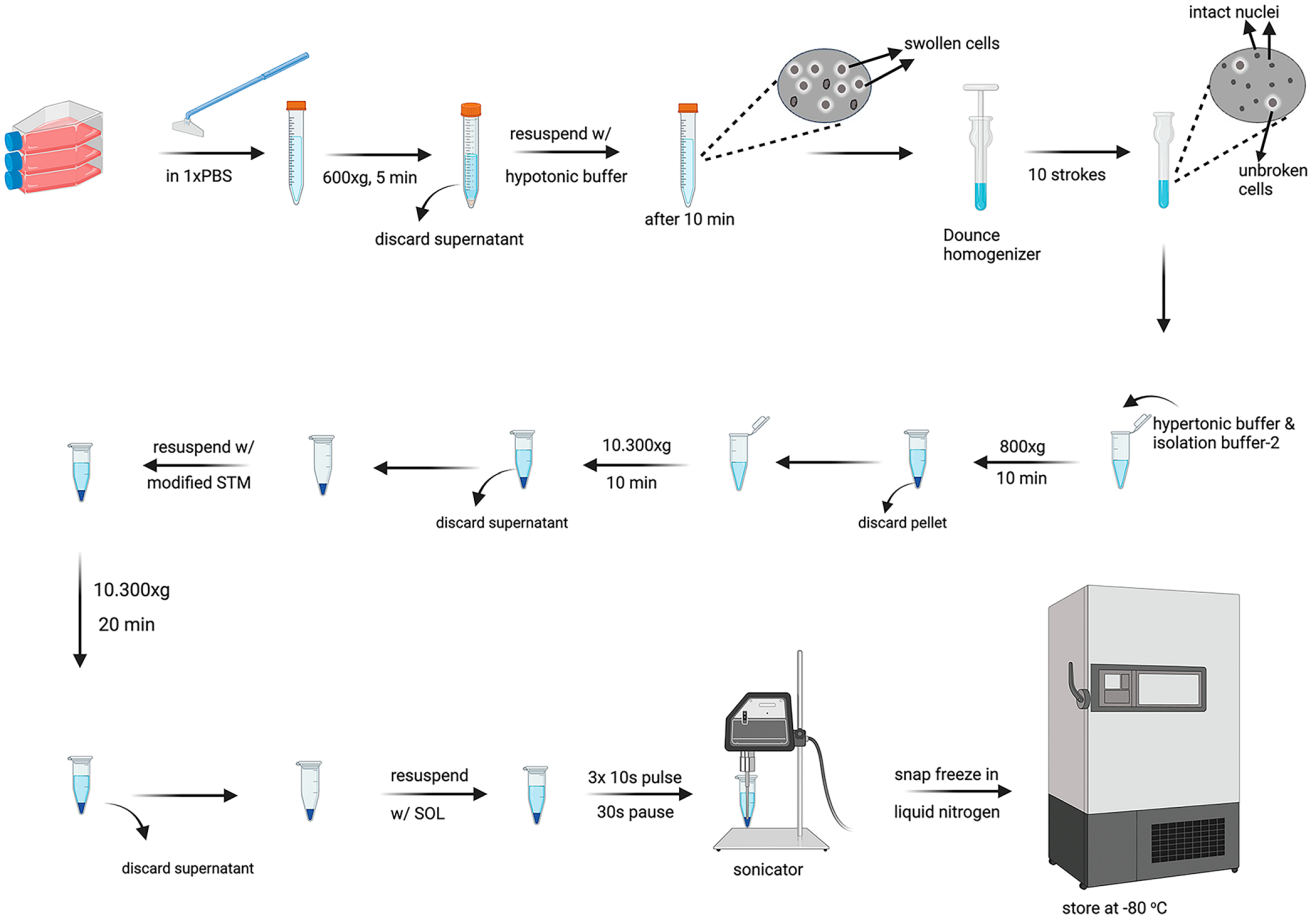
The workflow of hypotonic swelling method. Created in BioRender.com.

Cells grown in three T75 flasks were washed with 1× PBS and removed from the flasks using a cell scraper in 2 mL of ice‐cold PBS and transferred to 15 mL tubes. 3 mL of cold PBS was added to the flask to collect the remaining cells, which were then transferred to the same tube. The cells were centrifuged at 4°C at 600 × *g* for 5 min. After centrifugation, the supernatant was removed, and the cell pellet was resuspended in hypotonic buffer at a ratio of 5 mL per 1 *g* of cells. After incubating on ice for 10 min, the cells observed to swell under the microscope were lysed by 10 strokes (up and down) of tight pestle in an ice‐cold Dounce homogeniser. At this stage, the cells were examined under the phase‐contrast microscope, and by counting the nacked nuclei and intact cells, it was confirmed that approximately 90% of the cells' plasma membranes were disrupted, but the nuclear integrity was preserved (Figure 1). After homogenization, 0.1 mL of hypertonic buffer per 1 mL cell suspension was added. The entire sample was transferred to a 1.5 mL eppendorf tube, and Isolation buffer‐2 was added at twice the volume of the cell suspension. After centrifuged at 4°C at 800 × *g* for 10 min to precipitate cell debris, nuclei, and unbroken cells, the supernatant was transferred to a new tube and centrifuged at 4°C at 10,300 × *g* for 10 min to precipitate the mitochondrial fraction. The pellet was dissolved in Isolation buffer‐2 and centrifuged at 4°C at 10,300 × *g* for 20 min. After centrifugation, the supernatant was completely removed, and the pellet containing mitochondria was dissolved in SOL buffer. The buffers used in mitochondria isolation are provided in Table 1. The mitochondrial fraction was sonicated 3 times with 10 s pulses and 30 s pauses at 20 kHz and 20% amplitude, and mitochondrial proteins were quickly frozen in liquid nitrogen and stored at −80°C. The workflow of the hypotonic swelling method is shown in Figure 2.

### 2.5 Western Blot

In order to check the purity of the isolated mitochondrial fraction, western blot was performed by using mitochondrial (anti‐translocase of outer membrane 20 (TOM20)), cytoplasmic (anti‐ß‐tubulin), and nuclear (anti‐Histone H3) markers. The total extracted protein was quantified using the Pierce Bicinchoninic Acid (BCA) Protein Assay Kit (Thermo Scientific) and 30 μg of proteins were loaded into a 15% sodium dodecyl sulphate (SDS) containing polyacrylamide gel electrophoresis (PAGE) and transferred onto a polyvinylidene difluoride (PVDF) membrane (Millipore). Subsequently, the membrane was subjected to blocking for 1 h at room temperature, then incubated with a primary antibody overnight at 4°C, followed by a horse radish peroxidase (HRP)‐conjugated secondary antibody for 1 h at room temperature. Antibodies and dilutions utilised for western blot are as follows: TOM20 (Cell Signaling, 42,406) (1:1000), Histone H3 (Cell Signaling, 9715) (1:1000), ß‐tubulin (Sigma, T5201) (1:3000), HRP‐conjugated goat anti‐rabbit IgG (H + L) (Invitrogen) (1:5000), and HRP‐conjugated goat anti‐mouse IgG (H + L) (Invitrogen) (15000). Protein bands were visualised using the Super Signal West Femto Maximum Sensitivity Substrate (Thermo Scientific) via the GeneGnome5 Chemiluminescence imaging system (Syngene). The band intensities were quantitatively analysed using ImageJ (version 2.9.0/1.54f, NIH).

### 2.6 Assessment of the Efficacy of Mitochondrial Isolation Techniques

The efficacy of the mitochondrial isolation methods was systematically assessed based on the yield and purity of the resultant fractions. The total protein yield was quantified by measuring the protein concentration within the isolated fraction. While the mitochondrial enrichment capacity of the isolation method was assessed by calculating the Mitochondrial Enrichment Index (MEI), the purity of isolated fractions was evaluated utilising two distinct additional indices that were established for this purpose: the Cytosol Removal Index (CRI) and the Nuclei Removal Index (NRI). MEI was determined by calculating the ratio of the band intensity of the mitochondrial marker TOM20 in the mitochondrial fraction to that in the total cell lysate. CRI was ascertained by dividing the band intensity of the mitochondrial marker TOM20 by the band intensity of the cytoplasmic marker β‐tubulin within the mitochondrial fraction. Lastly, NRI was derived by dividing the band intensity of the mitochondrial marker TOM20 by the band intensity of the nuclear marker Histone H3 within the mitochondrial fraction. All experiments were repeated independently at least three times, and the average of the data was recorded.

### 2.7 Liquid Chromatography–Tandem Mass Spectrometry (LC–MS/MS) Analysis

#### 2.7.1 Peptide Preparation for MS

A slightly modified filter‐aided sample preparation (FASP) protocol was performed for the preparation of mitochondrial protein samples [22].

#### 2.7.2 LC‐Parameters

All peptide separations were carried out on a Thermo Scientific Ultimate 3000 Series RSLC nano pump coupled with a Thermo Scientific Ultimate 3000 Series TCC‐3000RS column compartments and a Thermo Fisher Scientific UltiMate 3000 Series RS autosampler controlled by Xcalibur 4.0 Software (Thermo Fisher Scientific, USA). For each analysis, the sample was loaded into Acclaim PepMap trapping column (5 mm × 300 μm i.d., C18, 5 μm, 100 Å) (Thermo Scientific, US) at 5 μL/min with aqueous solution containing 1% acetonitrile and 0.05% trifluoroacetic acid. The trapping column was put on‐line with Acclaim PepMap RSLC analytical column (15 cm × 75 μm i.d., C18, 2 μm, 100 Å) (Thermo Scientific, US) after 3 min. Peptide elution was performed by applying a mixture of mobile phase A (HPLC grade water with 0.1% formic acid) and B (HPLC grade acetonitrile with 0.1% formic acid). The cleaved samples were separated using a 130 min gradient elution at a flow rate of 0.30 μL/min, which directly interfaced with a Thermo Q Exactive mass spectrometer. The applied gradient steps were as follows: 0–45 min linear increase from 6% to 20% B; 45–75 min linear increase to 40% B; 75–90 in linear increase to 90%B and hold 30 min; 120–125 decreasing to 6% B; and 125–130 min 94% A. 2 μL of each sample was injected after the column temperature was set at 40°C. In positive mode, a heated electrospray ionisation source (HESI) was used to ionise the target compounds (the ionisation voltage: +2.3 kV; the capillary temperature: 300°C; the vaporizer temperature: 300°C; sheath gas and auxiliary gas flow were 50 and 30 arbitrary units, respectively; S lens RF level: 50).

#### 2.7.3 Data‐Dependent Acquisition

For data‐dependent acquisitions using the Q Exactive, the scan sequence started with an Orbitrap Full MS spectrum, configured with these parameters: a resolution of 70,000, a scan range of 400–2000 *m*/*z*, an automatic gain control (AGC) target of 3 × 10^6^, a maximum injection time of 60 ms, and centroid spectrum data type. The top 10 precursors were selected for MS^2^ analysis, performed with high‐energy collision dissociation (HCD) using the following settings: a resolution of 17,500, an AGC target of 1 × 10^5^, a maximum injection time of 100 ms, an isolation window of 2 *m*/*z*, a normalised collision energy (NCE) of 27, and data acquired as a centroid spectrum. External calibration was performed by calibration solution (LTQ Velos ESI Positive Ion Calibration Solution 88,323, Pierce, USA) in positive modes before each sample series. The underfill ratio was adjusted to 9%, equating to an intensity threshold of 1.5 × 10^5^. Furthermore, unassigned and singly charged species were omitted from MS^2^ analysis, with dynamic exclusion configured to automatic.

#### 2.7.4 MS Data Analysis

Each sample was run three times by MS. Raw data was processed by using Proteome Discoverer 2.2 (Thermo Fisher Scientific, USA) with specific parameters (peptide mass tolerance: 10 ppm, MS/MS mass tolerance: 0.02 Da, mass precision: 2 ppm, minimal peptide length: 6 amino acids, carbamidomethylation of cysteine (+57.021464): fixed modification, the oxidation of methionine (+15.994915) and deamidation of asparagine (0.984016): variable modification). For each protein, a few identified unique peptides were set to two. The input data were searched against the UniProtKB‐SwissProt database, and identified proteins were grouped based on biological process using the Gene Ontology (GO) database.

### 2.8 Statistical Analysis

Differences between data sets were evaluated statistically by using the One‐way ANOVA test in the GraphPad Prism 9.0.0 software (Graph‐Pad Software Inc., San Diego, CA). Data were presented as mean ± standard deviation. A *p*‐value less than 0.05 was accepted as statistically significant.

## 3 Results

This study applied four different methods to isolate pure mitochondria from primary human skeletal myoblasts. All of these methods provide mitochondrial isolation solely for proteomic studies, and no mitochondrial respiration tests have been conducted on the isolated mitochondria by these protocols. Qproteome Mitochondria Isolation Kit (Qiagen) offers short and long‐isolation methods that provide standard and high‐purity mitochondria. Although both protocols were applied in our study, mitochondria pellet was observed only with the short isolation protocol. Mitochondria Isolation Kit for Cultured Cells (Thermo Scientific) also offers two options for isolation. Mitochondria isolation with Dounce homogenization by 70 strokes (up and down) was applied, and a mitochondria pellet was obtained.

**FIGURE 3:**
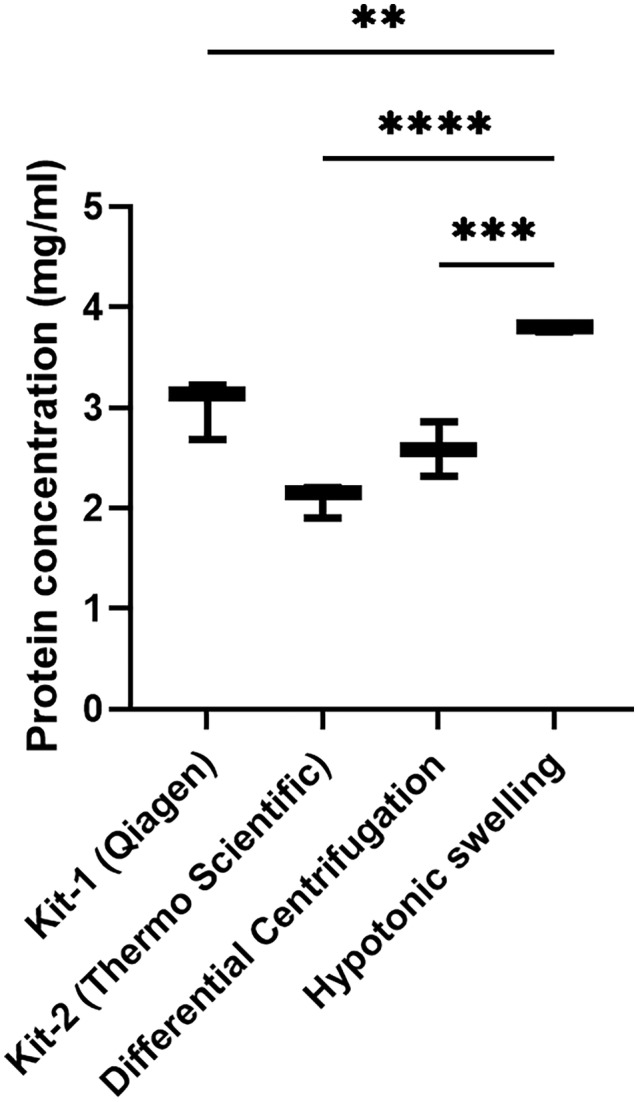
The graph showing the results of comparison of total protein yield in mitochondrial fractions isolated with four different mitochondria isolation methods. Differences between data sets were assessed by One‐way ANOVA test with **p < 0.01, ***p < 0.001, ****p < 0.0001.

The effectiveness of these two kit‐based and two manual methods was evaluated based on the isolated fraction's total protein yield, enrichment capacity and purity. While mitochondrial pellets were successfully obtained by all four methods, yield, enrichment capacity and purity of the fraction differed. Statistical analysis showed that there is a significant difference between the total protein concentration of hypotonic swelling methods and that of other methods. The highest protein yield was achieved using the hypotonic swelling method, as shown in Figure 3.

**FIGURE 4:**
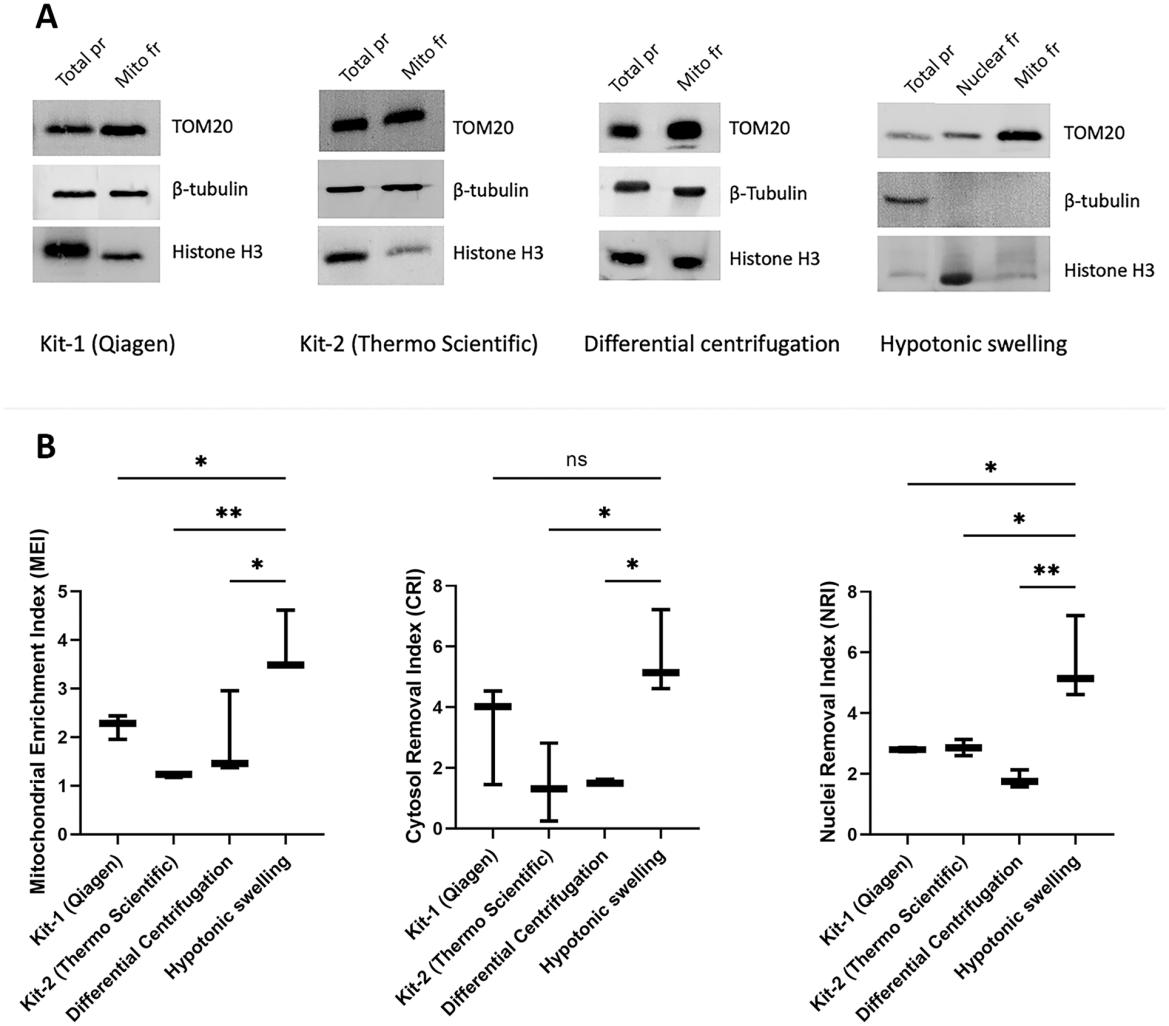
(A) Comparison of the purity of mitochondrial fractions isolated with four different mitochondria isolation methods by western blot. 30 μg of total protein was loaded, and blots were probed with anti‐TOM20, Histone H3 and ß‐tubulin antibodies specific for mitochondrial, nuclear and cytoplasmic fractions, respectively. (B) The graph showing the results of comparison of mitochondrial enrichment capacity and the purity of the mitochondrial fractions isolated with four different mitochondria isolation methods. Differences between data sets were assessed by One‐way ANOVA test with **p < 0.01, ***p < 0.001. CRI, Cytosol Removal Index; MEI, Mitochondrial Enrichment Index; NRI, Nuclei Removal Index.

Western blot was performed with antibodies specific to different cellular compartments to check the purity of different mitochondria isolation methods. As seen in Figure 4, it was determined that the mitochondria isolation method with the least nuclear contamination and no cytoplasmic contamination was the hypotonic swelling method. The purity of the isolated mitochondrial fraction was analysed by calculating the band intensities in these blots. The highest value was obtained from the hypotonic swelling method when comparing isolation methods regarding the MEI. Similarly, the highest CRI and NRI values, which indicate how effectively the cytoplasm and nucleus, respectively, were removed from the isolated mitochondrial fraction, were obtained by the hypotonic swelling method. Based on statistical analysis, it was decided that the most suitable method for isolating mitochondria from primary human skeletal myoblasts is the hypotonic swelling method (Figure 4).

**FIGURE 5:**
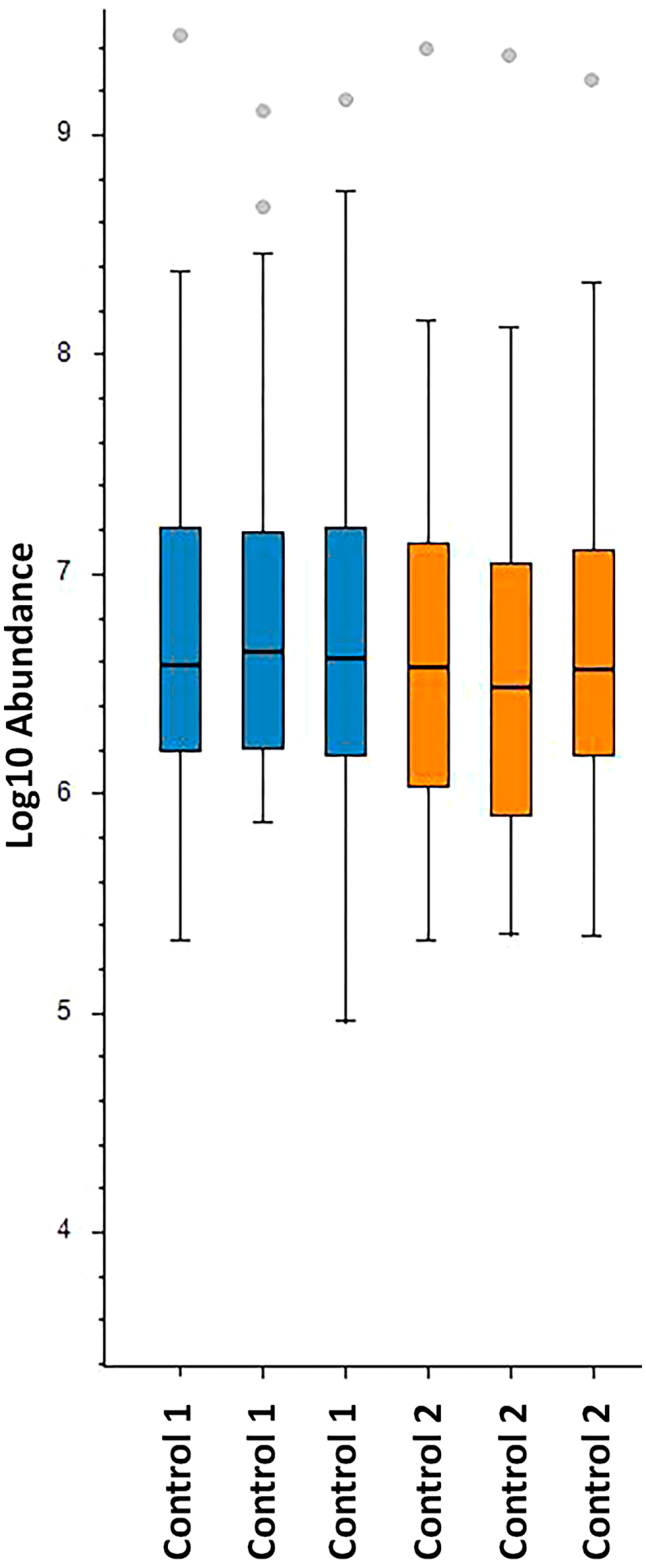
Sample abundance graph of mitochondrial proteins detected by LC–MS/MS analysis of mitochondrial fractions isolated from primary skeletal myoblasts by the hypotonic swelling method. Each sample was run three times by MS. The median values are represented by a horizontal line within each box, while points positioned outside the boxes are identified as outliers. (Control 1: 8063; Control 2: NH11‐1462A).

**FIGURE 6:**
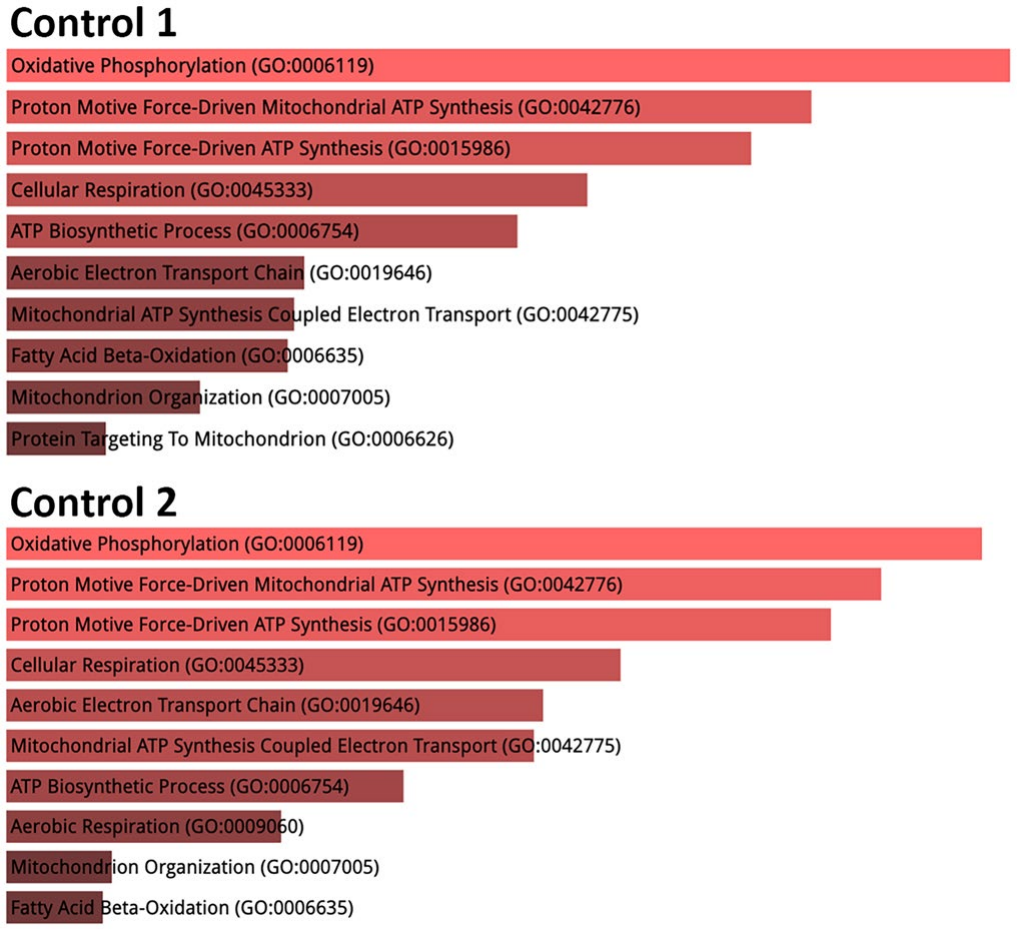
Gene ontology (GO) enrichment analysis of the mitochondrial gene based on the biological process category. Bar graphs are sorted by *p* value (*p* < 0.05). The longer bars and lighter coloured bars mean that the gene set is more significant.

Mitochondrial fraction proteins isolated from control primary skeletal myoblasts by the hypotonic swelling were subjected to label‐free LC–MS/MS analysis to test its effectiveness for proteomic analyses. Each protein sample was analysed in triplicate. Peak intensities were utilised for density computation during the analysis. The identified proteins were examined against the human reference proteome (Proteome ID: UP000005640, reviewed 20.418) obtained from the Uniprot database and the human mitochondrial proteome (GO: 0005739, 1667). Following the LC–MS/MS analysis of the mitochondrial proteome of primary skeletal myoblasts, an average of 100 mitochondrial proteins were confidently identified. The sample abundance graph and the list of identified mitochondrial proteins are presented in Figure 5 and Table S1, respectively. Moreover, gene ontology enrichment analysis based on the biological processes revealed that oxidative phosphorylation, proton motive force‐driven mitochondrial ATP synthesis, and cellular respiration are enriched (Figure 6).

## 4 Discussion

Primary skeletal muscle cell cultures serve as crucial representatives of disease models [23, 24, 25, 26]. They provide the most biologically precise data and more accurately reflect in vivo compared to immortalised cell lines [27, 28]. Although primary skeletal muscle cells present several challenges, including difficulties in culturing, transfection and general handling, there is a growing preference for using them due to ethical considerations associated with animal experiments.

Given their intrinsic characteristics, skeletal muscle cells require higher energy demand, making mitochondria important to their function. For this reason, elucidating the mitochondrial proteome in skeletal muscle cells is vital for many muscle disorders, especially those involving mitochondrial dysfunction. Since 99% of mitochondrial proteins are synthesised on free ribosomes in the cytosol and then transported to mitochondria, these proteins can be found in both cytosol and mitochondria. Therefore, it is necessary to obtain pure mitochondria for proteomic analyses to identify only mitochondria‐located proteins. The importance of mitochondrial proteomic studies, which reveal dynamic proteome changes in response to physiological, pathological and environmental conditions in skeletal muscle cells, cannot be underestimated. This knowledge is critical for unravelling the molecular basis of muscle function, identifying novel therapeutic targets for muscle diseases, and understanding the mechanisms of muscle ageing and regeneration.

In the literature, mitochondria isolation from various biological sources is performed for different purposes, such as high‐throughput omics analyses or mitochondrial function tests. Also, many different methods have been described for mitochondria isolation. Some studies use mitochondrial isolation kits, while others employ differential or density‐gradient centrifugation methods [11, 15, 29, 30, 31]. The choice of isolation method depends on the specific requirements of the study. Although these methods have their advantages and disadvantages, not every isolation method is suitable for every cell/tissue type. For instance, differential centrifugation is suitable for studies that require large quantities of mitochondria and where some cellular contamination can be tolerated. In studies where the purity and integrity of mitochondria are crucial, density gradient centrifugation is preferred [32]. However, it has been indicated that approximately 1 × 10^9^ cells (about 200–300 T75 flasks) are required in this method due to a significant amount of protein loss during the mitochondrial purification process [14]. Therefore, primary cells having a limited proliferation capacity (Hayflick limit) [20] allow isolation from fewer cells, and the density gradient centrifugation method cannot be used in these cells. Additionally, the choice of isolation method may vary depending on the type of experiment to be performed after isolation. Although it is not necessary to preserve mitochondrial viability and respiratory capacity in proteomic studies, removing other cellular compartments, such as cytoplasm and nucleus, from the mitochondrial fraction is important.

In this study, mitochondria isolation experiments were conducted using four different methods, including two commercially available isolation kits (Qproteome Mitochondria Isolation Kit‐Qiagen & Mitochondria Isolation Kit for Cultured Cells‐Thermo Scientific) and two manual methods (differential centrifugation and hypotonic swelling). Although these four methods are basically based on differential centrifugation, they differ in their isolation buffer, cell homogenization methods, centrifuge speed, and durations.

Studies in the literature have successfully isolated mitochondria in different tissues and cell lines using commercial kits and the differential centrifugation method [11, 15, 29, 33, 34, 35, 36, 37, 38]. However, in our study, a pure mitochondrial fraction could not be obtained from primary skeletal myoblasts with any of the kits we used or the differential centrifugation method due to cytoplasmic and nuclear contamination. Based on the overall yield, MEI, CRI and NRI values, the hypotonic swelling method is recommended as the most appropriate technique for isolating pure mitochondria from primary skeletal myoblasts. This technique includes additional steps in which cells swell in a hypotonic environment before homogenization, allowing for easier separation of cellular fractions, thus minimising cellular contamination. Panov et al. applied the hypotonic swelling method to isolate mitochondria from human lymphoblastoid cells derived by transforming peripheral blood leukocytes with the Epstein–Barr virus [37]. Although hypotonic and hypertonic buffers are the same as those in our method, our isolation buffer content, the number of strokes (up and down) of the Dounce homogeniser, centrifuge speed and durations differ.

Many studies in which mitochondria are isolated from cell cultures use a cell culture medium containing antibiotics to control bacterial contamination. However, it is known that antibiotics belonging to the aminoglycoside group, such as gentamicin, reduce the respiratory capacity of mitochondria [39, 40]. The use of antibiotics in gene expression studies is not recommended because they not only disrupt mitochondrial function but also change the gene expression patterns of cells [41]. Since we aimed to perform proteomic analysis on the isolated mitochondrial fraction, we did not add antibiotics to the cell culture medium.

Although the exact buffer contents of the commercial isolation kits are unknown, buffers used for manual methods are comparable. While Tris‐based buffer is used in the differential centrifugation method, MOPS‐based buffer has been utilised in the hypotonic swelling method. Additionally, the isolation buffer in the differential centrifugation method contains high mannitol and low sucrose, whereas the buffer in the hypotonic swelling method comprises high sucrose and low mannitol. In the literature, it is observed that many different buffer contents are used for mitochondrial isolation. In some studies, high concentrations of sucrose and low concentrations of mannitol are used, as in our study [13, 35], while in others, high concentrations of mannitol and low concentrations of sucrose are used [36, 42]. However, due to its osmotic activity and ability to penetrate the mitochondrial membrane, it is recommended not to use high concentrations of mannitol for mitochondrial isolation. Instead, isolation buffers containing high concentrations of sucrose are suggested [40]. Additionally, it is seen in the literature that fat‐free BSA is used in some of the mitochondrial isolation buffers according to downstream applications [13, 43, 44, 45, 46, 47]. Fat‐free BSA is known to capture fatty acids released when the cell is disrupted, prevent mitochondrial damage and essential for oxidative phosphorylation [45, 46]. Therefore, it is recommended to use BSA in isolation buffers when isolating mitochondria for functional analyses such as respiratory assays. However, since BSA might complicate proteomics analyses by masking mitochondrial proteins, its use is not recommended in mass spectrometry analysis [45].

In our study, LC–MS/MS analysis performed after isolation of pure mitochondrial fraction from primary skeletal myoblasts covered almost 10% of the total mitochondrial proteome. Due to the limited proliferation capacity of primary cells, mitochondrial isolation has been possible from a more limited number of cells than skeletal muscle tissue and myoblast cell lines. Therefore, the limited amount of protein loaded into the instrument for LC–MS/MS analysis allowed the identification of 100 high‐confidence mitochondrial proteins. In one study, mitochondria were isolated from 70 times more cells than in our study, and four times more mitochondrial proteins were identified as a result of LC–MS/MS analysis compared to our study [48]. Compared to the results of this study, it is thought that the amount of protein we identified by LC/MS–MS may be sufficient to identify differentially expressed mitochondrial proteins. It is understood that the amount of protein loaded into the LC–MS/MS instrument is directly proportional to the number of proteins identified. In the literature, mitochondrial proteomic studies are conducted with skeletal muscle tissue and myoblast cell lines [8, 49]. However, to our knowledge, our study is the first mitochondrial proteomic study in which purity was checked after the isolation of mitochondria from primary myoblasts. Gene ontology analysis showed that identified proteins are mainly associated with mitochondrial energy metabolism and biogenesis, as expected. Morgenstern et al. revealed that approximately 60% of yeast mitochondrial proteome contain proteins involved in the respiratory chain, energy metabolism and mitochondrial biogenesis [50]. Considering the low peptide coverage of our study, it is expected that some of the mitochondrial proteins can't be detected. This issue results from working with the low number of primary cells with the limited proliferation capacity.

The limitation of this study can be considered as the lack of functional tests of the mitochondria. Since we disrupted mitochondrial integrity by using SOL buffer containing non‐ionic detergent and by sonication during the extraction of the mitochondrial proteins, it is impossible to measure the respiratory capacity of the isolated mitochondria. Furthermore, for the mitochondria to maintain their respiratory capacity, the mitochondrial isolation buffer needs to contain BSA. However, adding BSA to the isolation buffer is not recommended in proteomic studies because it interferes with protein quantification assays, creates noise and reduces total protein coverage [45]. Since we performed proteomic studies on isolated mitochondria, we couldn't conduct mitochondrial function tests. Our method is sufficient from a proteomic analysis perspective, and can also be tested in respiratory studies by using different buffers in further studies. Krakowczyk et al. performed both proteomics and respirometry analysis simultaneously; however, they used different mitochondria isolation buffers [51]. Moreover, the coverage of mitochondrial proteomics was low due to the low number of cells used for mitochondria isolation. This is an inevitable consequence of working with primary human cells, considering the difficulty of accessing a biopsy and ethical concerns, but it is still possible to detect mitochondrial proteins that exhibit quantitative differences. Especially with the advancement of proteomic analysis techniques, it will be possible to identify more proteins with less protein input [7, 52].

There are many different mitochondria isolation methods in the literature; however, finding the one best suited to the examined cell type may take a long time and effort. This study has demonstrated that the hypotonic swelling method is the best for primary skeletal myoblasts and will serve as a valuable reference point for those working with primary skeletal muscle cells. By optimising the mitochondrial isolation process for proteomic studies, our work will not only improve the methodological framework but also significantly contribute to the broader understanding of skeletal muscle biology in terms of mitochondrial function and dynamics.

## Author Contributions

**Evrim Aksu‐Menges:** methodology (equal); writing – original draft (lead); writing – review and editing (equal); investigation (lead); formal analysis (lead); visualization (lead); validation (lead). **Eray Taha Kumtepe:** investigation (supporting); visualization (supporting); validation (supporting). **Gurler Akpinar:** data curation (lead); writing – original draft (supporting); investigation (supporting). **Burcu Balci‐Hayta:** project administration (lead); supervision (lead); methodology (equal); writing – review and editing (equal).

## Ethics Statement

In this study, the experimental protocol was approved by the Hacettepe University Faculty of Medicine Ethical Review Board (2020/07‐12).

## Conflicts of Interest

The authors declare no conflicts of interest.

## Supporting information


**Table S1:** The list of identified mitochondrial proteins detected by LC–MS/MS analysis of mitochondrial fractions isolated from primary skeletal myoblasts by the hypotonic swelling method.

## Data Availability

The data that supports the findings of this study are available in the Supporting Information of this article.
